# The Geometry of Generalized Likelihood Ratio Test

**DOI:** 10.3390/e24121785

**Published:** 2022-12-06

**Authors:** Yongqiang Cheng, Hongqiang Wang, Xiang Li

**Affiliations:** College of Electronic Science and Engineering, National University of Defense Technology, Changsha 410073, China

**Keywords:** composite hypothesis testing, generalized likelihood ratio test, maximum likelihood estimation, information geometry, statistical inference, information loss

## Abstract

The generalized likelihood ratio test (GLRT) for composite hypothesis testing problems is studied from a geometric perspective. An information-geometrical interpretation of the GLRT is proposed based on the geometry of curved exponential families. Two geometric pictures of the GLRT are presented for the cases where unknown parameters are and are not the same under the null and alternative hypotheses, respectively. A demonstration of one-dimensional curved Gaussian distribution is introduced to elucidate the geometric realization of the GLRT. The asymptotic performance of the GLRT is discussed based on the proposed geometric representation of the GLRT. The study provides an alternative perspective for understanding the problems of statistical inference in the theoretical sense.

## 1. Introduction

The problem of hypothesis testing under statistical uncertainty arises naturally in many practical contexts. In these cases, the probability density functions (PDFs) under either or both hypotheses need not be completely specified, resulting in the inclusion of unknown parameters in the PDFs to express the statistical uncertainty in the model. The class of hypothesis testing problems with unknown parameters in the PDFs is commonly referred to as composite hypothesis testing [1]. The generalized likelihood ratio test (GLRT) is one of the most widely used approaches in composite hypothesis testing [2]. It involves estimating the unknown parameters via the maximum likelihood estimation (MLE) to implement a likelihood ratio test. In practice, the GLRT appears to be asymptotically optimal in the sense of the Neyman–Pearson criterion and usually gives satisfactory results [3]. As the GLRT combines both estimation and detection to deal with the composite hypothesis testing problem, its performance, in general, will depend on the statistical inference performance of these two aspects. However, in the literature, there is no general analytical result associated with the performance of the GLRT [1].

In recent years, the development of new theories in statistical inference has been characterized by the emerging trend of geometric approaches and their powerful capabilities, which allows one to analyze statistical problems in a unified perspective. It is important to link the GLRT to the geometrical nature of estimation and detection, which provides a new viewpoint on the GLRT. The general problem of composite hypothesis testing involves a decision between two hypotheses where the PDFs are themselves functions of unknown parameters. One approach to the understanding of performance limitations of statistical inference is via the theory of information geometry. In this context, the family of probability distributions with a natural geometrical structure is defined as a statistical manifold [4]. Information geometry studies the intrinsic properties of statistical manifolds which are endowed with a Riemannian metric and a family of affine connections derived from the log-likelihood functions of probability distributions [5]. It provides a way of analyzing the geometrical properties of statistical models by regarding them as geometric objects.

The geometric theory of statistics was firstly introduced in the 1940s by Rao [6], where the Fisher information matrix was regarded as a Riemannian metric on the manifold of probability distributions. Then, in 1972, a one-parameter family of affine connections was introduced by Chentsov in [7]. Meanwhile, Efron [8] defined the concept of *statistical curvature* and discussed its basic role in the high-order asymptotic theory of statistical inference. In 1982, Amari [5,9] developed a duality structure theory that unified all of these theories in a differential-geometric framework, leading to a large number of applications.

In the area of hypothesis testing, the geometric perspectives have acquired relevance in the analysis and development of new approaches to various testing and detection contexts. For example, Kass and Vos [10] provided a detailed introduction to the geometrical foundations of asymptotic inference of curved exponential families. Garderen [11] presented a global analysis of the effects of curvature on hypothesis testing. Dabak [12] induced a geometric structure on the manifold of probability distributions and enforced a detection theoretic specific geometry on it, while Westover [13] discussed the asymptotic limit in the problems of multiple hypothesis testing from the geometrical perspective. For the development of new approaches to hypothesis testing, Hoeffding [14] proposed an asymptotically optimal test for multinomial distributions in which the testing can be denoted in terms of the Kullback–Leibler divergence (KLD) between the empirical distribution of the measurements and the null hypothesis, where the alternate distribution is unrestricted. In the aspect of signal detection, Barbaresco et al. [15,16,17] studied the geometry of Bruhat–Tits complete metric space and upper-half Siegel space and introduced a matrix constant false alarm rate (CFAR) detector which improves the detection performance of the classical CFAR detection.

As more and more new analyses and new approaches have benefited from the geometric and information-theoretic perspectives of statistics, it appears to be important to clarify the geometry of existing problems that is promising to gain new ways to deal with the statistical problems. In this paper, a geometric interpretation of the GLRT is sought from the perspective of information geometry. Two pictures of the GLRT are presented for the cases where unknown parameters are and are not the same under each hypothesis, respectively. Under such an interpretation, both detection and estimation associated with the GLRT are regarded as geometric operations on the statistical manifold. As a general consideration, curved exponential families [9], which include a large number of the most common used distributions, are taken into account as the statistical model of hypothesis testing problems. A demonstration of one-dimensional curved Gaussian distribution is introduced to elucidate the geometric realization of the GLRT. The geometric structure of the curved exponential families developed by Efron [8] in 1975 and Amari [9] in 1982 provides a theoretical foundation for the analysis. The geometric formulation of the GLRT presented in this paper makes it possible for several advanced notions and conclusions in the information geometry theory to be transferred and applied to the performance analysis of the GLRT.

The main contributions of this paper are summarized as follows:

- A geometric interpretation of the GLRT is proposed based on the differential geometry of curved exponential families and duality structure theory developed by Amari [9]. Two geometric pictures of the GLRT are presented in the theoretical sense, which provides an alternative perspective for understanding the problems of statistical inference.
- The asymptotic performance of the GLRT is discussed based on the proposed geometric representation of the GLRT. The information loss when performingthe MLE using a finite number of samples is related to the flatness of the submanifolds determined by the GLRT model.

In the next section, alternative viewpoints on the likelihood ratio test and the maximum likelihood estimation are introduced from the perspective of information theory. The equivalences between the Kullback–Leibler divergence, likelihood ratio test, and the MLE are highlighted. The principles of information geometry are briefly introduced in Section 3. In Section 4, the geometric interpretation of the GLRT is presented in consideration of the geometry of curved exponential families. We present an example of the GLRT where a curved Gaussian distribution with one unknown parameter is involved, and a further discussion on the geometry of the GLRT. Finally, conclusions are obtained in Section 5.

## 2. Information-Theoretic Viewpoints on Likelihood Ratio Test and Maximum Likelihood Estimation

In statistics, the likelihood ratio test and maximum likelihood estimation are two fundamental concepts related to the GLRT. The likelihood ratio test is a very general form of testing model assumptions, while the maximum likelihood estimation is one of the most common approaches to parameter estimation. Both of them are associated with the Kullback–Leibler divergence [18], which is equivalent to the relative entropy [19] in information theory.

For a sequence of observations $x={\left(x_{1},x_{2},\ldots ,x_{N}\right)}^{T}\in R^{N}$ which is independently and identically distributed (i.i.d.), the binary hypothesis testing problem is used to decide whether this sequence x originates from the null hypothesis $H_{0}$ or the alternative hypothesis $H_{1}$ with probability distributions $p_{0}\left(x\right)$ and $p_{1}\left(x\right)$, respectively. The likelihood ratio is given by

(1) $$L=\frac{p_{1}\left(x\right)}{p_{0}\left(x\right)}=\prod_{i=1}^{N}\frac{p_{1}\left(x_{i}\right)}{p_{0}\left(x_{i}\right)}$$

Assume q(x) is the empirical distribution (frequency histogram acquired via Monte Carlo tests) of observed data. For large *N*, in accordance with the strong law of large numbers [20], the log likelihood ratio test in the Neyman–Pearson formulation $\ln L\underset{H_{0}}{\overset{H_{1}}{≷}}\gamma$ is equivalent to

(2) $$D\left(q∥p_{0}\right)-D\left(q∥p_{1}\right)\underset{H_{0}}{\overset{H_{1}}{≷}}\frac{1}{N}\gamma ≜\gamma^{\prime }$$

where $\underset{H_{0}}{\overset{H_{1}}{≷}}$ denotes that the test is to decide $H_{1}$ if “>” is satisfied, or to decide $H_{0}$, and vice versa. The quantity

(3) $$D\left(q∥p\right)=\int_{R^{N}}q\left(x\right)\ln\frac{q(x)}{p(x)}dx$$

is the KLD from q(x) to p(x). Note that *x* is dropped from the notion *D* for simplifying the KLD expression without confusion.

Equation (2) indicates that the likelihood ratio test is equivalent to choosing the hypothesis that is “closer” to the empirical distribution in the sense of the KLD. The test can be referred to as a generalized minimum dissimilarity detector in a geometric viewpoint.

Now, consider another, slightly different problem where the observations x are from a statistical model represented by p(x|θ) with unknown parameters θ. The problem is to estimate the unknown parameters θ based on observations x. The likelihood function for the underlying estimation problem is

(4) $$p\left(x|\theta \right)=\prod_{i=1}^{N}p\left(x_{i}|\theta \right)$$

In a similar way, for large *N*, maximizing the likelihood (4) to find the maximum likelihood estimate of θ is equivalent to finding θ, which minimizes the KLD $D(q∥p_{\theta })$, i.e.,

(5) $$\hat{\theta }=\arg\min_{\theta }D\left(q∥p_{\theta }\right)$$

where $p_{\theta }$ is used as a surrogate for p(x|θ).

The above results provide an information-theoretic view to the problem of hypothesis testing and maximum likelihood estimation in statistics. From the perspective of information difference, these results have profound geometric meanings and can be geometrically analyzed and viewed in the framework of information geometry theory, from which additional insights into the analysis of these statistical problems, as well as their geometric interpretations, are obtained.

## 3. Principles of Information Geometry

### 3.1. Statistical Manifold

Information geometry studies the natural geometric structure of the parameterized family of probability distributions S={p(x|θ)} specifying by a parameter vector $\theta :=[\theta_{1},\ldots ,\theta_{n}]$, in which x is the samples of a random variable X. When the probability measure on the sample space is continuous and differentiable and the mapping θ↦p(x|θ) is injective [5], the family *S* is considered as a statistical manifold with θ as its coordinate system [4].

**Figure 1:**
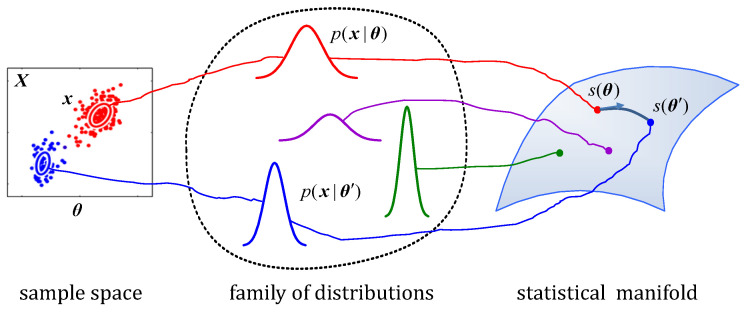
Diagram of a statistical manifold. θ and $s(\theta^{\prime })$ denote parameters of the family of distributions from different samples X. The connection on the statistical manifold *S* represents a geodesic (the shortest line) between points s(θ) and $s(\theta^{\prime })$. The length of the geodesic serves as a distance measure between two points on the manifold. The arrow on the geodesic starting from the point s(θ) denotes the tangent vector, which gives the direction of the geodesic.

Figure 1 demonstrates the diagram of a statistical manifold. For a given parameter vector $\theta \in \Theta \subset R^{n}$, the measurement x in the sample space X is an instantiation of a probability distribution p(x|θ). Each p(x|θ) in the family of distributions is specified by a point s(θ) on the manifold *S*. The *n*-dimensional statistical manifold is composed of the parameterized family of probability distributions S={p(x|θ)} with θ as a coordinate system of *S*.

Various families of probability distributions correspond to specific structures of the statistical manifold. Information geometry takes the statistical properties of samples as the geometric structure of a statistical manifold, and utilizes differential geometry methods to measure the variation of information contained in the samples.

### 3.2. Fisher Information Metric and Affine Connections

The metric and connections associated with a manifold are two important concepts in information geometry. For a statistical manifold consisting of a parameterized family of probability distributions, the Fisher information matrix (FIM) is usually adopted as a Riemannian metric tensor of the manifold [6], which is defined by the inner product between tangent vectors at a point on the manifold. It is denoted by $G\left(\theta \right)=[g_{ij}\left(\theta \right)]$, where

(6) $$g_{ij}\left(\theta \right)=E\left\{\frac{\partial \log p(x|\theta )}{\partial \theta_{i}}\cdot \frac{\partial \log p(x|\theta )}{\partial \theta_{j}}\right\}$$

where $\left\{\partial \log\left(\cdot \right)/\partial \theta_{i}\right\}$ is considered as a basis for the vector space of random variable X. The tangent space of *S* at θ, denoted as $T_{\theta }\;\left(S\right)$, is identified as the vector space. Based on the above definition, the FIM metric determines how the information distance is measured on the statistical manifold.

When considering the relationships between two tangent spaces $T_{\theta }$ and $T_{\theta +d\theta }$ at two neighboring points θ and θ+dθ (*d* is the differential operator), an affine connection is defined by which the two tangent spaces become feasible for comparison. When the connection coefficients are all identically 0, then *S* is flat manifold that “locally looks like” a Euclidean space with zero curvatures everywhere. The most commonly used connection is called α-connections [9],

(7) $${\overset{\alpha }{\Gamma }}_{jim}\left(\theta \right)=E_{\theta }\left\{\partial_{j}\partial_{i}\;l\left(x,\;\theta \right)\;\partial_{m}l\left(x,\;\theta \right)\right\}+\frac{1-\alpha }{2}E_{\theta }\left\{\partial_{j}l\left(x,\;\theta \right)\;\partial_{i}l\left(x,\;\theta \right)\;\partial_{m}l\left(x,\;\theta \right)\right\}$$

where ${\overset{\alpha }{\Gamma }}_{jim}$ denotes the connection coefficients with i,j,m=1,…,n, and

(8) $$\partial_{i}=\frac{\partial }{\partial \theta_{i}}\;\mathrm{and}\;l\left(\theta ,\;x\right)=\log p\left(x|\theta \right).$$

In (7), α=0 corresponds to the Levi–Civita connection, while α=1 defines the *e*-connection and α=−1 defines the *m*-connection. Under the *e*-connection and *m*-connection, an exponential family with natural parameter θ coordinate and a mixture family with expectation parameter η coordinate are both flat manifolds [9]. Statistical inference with respect to the exponential family greatly benefits from the geometric properties of the flat manifold. By using the methods of differential geometry, many additional insights into the intrinsic structure of probability distributions can be obtained, which opens a new perspective on the analysis of statistical problems. In the next section, a geometric interpretation of the GLRT and further discussions are sought based on the principles of information geometry.

## 4. Geometry of the Generalized Likelihood Ratio Test

As a general treatment, the curved exponential families, which encapsulate many important distributions for real-world problems, are considered as the statistical model for the hypothesis testing problems discussed in this paper. In this section, the MLE solution to parameter estimation for curved exponential families is derived. We then present two pictures of the GLRT, which are sketched based on the geometric structure of the curved exponential families developed by Efron [8] in 1975 and Amari [9] in 1982, to illustrate the information geometry of the GLRT. An example of the GLRT for a curved Gaussian distribution with a single unknown parameter is given, which is followed by a further discussion on the geometric formulation of the GLRT.

### 4.1. The MLE Solution to Statistical Estimation for Curved Exponential Families

Exponential families contain lots of the most commonly used distributions, including the normal, exponential, Gamma, Beta, Poisson, Bernoulli, and so on [21]. The curved exponential families are the distributions whose natural parameters are nonlinear functions of “local” parameters. The canonical form of a curved exponential family [9] is expressed as

(9) $$p\left(x|u\right)=\exp\left\{C\left(x\right)+\theta^{T}\left(u\right)F\left(x\right)-\varphi \left(\theta (u)\right)\right\}=p\left(x|\theta (u)\right)$$

where x∈X is a vector of samples, $\theta :=[\theta_{1},\ldots ,\theta_{n}]$ are the natural parameters, $u\in R^{m}\left(m<n\right)$ are local parameters standing for the parameters of interest to be estimated, which is specified by (9), while $F\left(x\right):={\left[F_{1}\left(x\right),\cdots ,F_{n}\left(x\right)\right]}^{T}$ denote sufficient statistics with respect to $\theta =(\theta_{1},\cdots ,\theta_{n})$, which take values from the sample space *X*. φ is the potential function of the curved exponential family and it is found from the normalization condition $\int_{X}p\left(x|\theta \right)dx=1$, i.e.,

(10) $$\varphi \left(\theta \right)=\log\int_{X}\exp\left\{C\left(x\right)+\sum_{i}^{n}\left(\theta_{i}F_{i}\left(x\right)\right)\right\}dx$$

The term “curved” is due to the fact that the curved exponential family in (9) is a submanifold of the canonical exponential family p(x|θ) by the embedding u⟶θ(u).

Let l(θ,x)=logp(x|θ) be the log-likelihood and $\nabla_{u}\theta$ be the Jacobian matrix of the natural parameter θ. According to (9),

(11) $$\begin{array}{cc} \nabla l(\theta ,\;x) & =\nabla \left\{\theta^{T}\left(u\right)F\left(x\right)-\varphi \left(\theta (u)\right)\right\}\\ \; & =\nabla \theta^{T}\left(u\right)\left[F(x)-\eta (u)\right] \end{array}$$

where η(u) is the expectation of the sufficient statistics F(x), i.e.,

(12) $$\eta \left(u\right):=E_{p(\cdot ,u)}\left\{F\left(x\right)\right\}$$

and is called the expectation parameter, which defines a distribution of mixture family [4]. The natural parameter θ(u) and expectation parameter η(u) are connected by the Legendre transformation [9], as

(13) $$\eta =\nabla_{\theta }\varphi \left(\theta \right),\;\theta =\nabla_{\eta }\phi \left(\eta \right)$$

where ϕ(η) is defined by

(14) $$\phi \left(\eta \right)=\max_{\theta }\left\{\eta^{T}\theta -\varphi \left(\theta \right)\right\}$$

Therefore,

(15) $$\nabla_{u}\varphi \left(u\right)=\nabla_{u}\theta^{T}\left(u\right)\nabla_{\theta }\varphi \left(\theta \right)=\nabla_{u}\theta^{T}\left(u\right)\eta \left(u\right)$$

Thus, the maximum likelihood estimator $\hat{u}$ of the local parameter in (9) can be obtained by the following likelihood equation:

(16) $$\nabla_{u}l\left(\hat{u}\right)=\nabla_{u}\log p\left(x|u\right)=\nabla_{u}\theta^{T}\left(\hat{u}\right)\left[F\left(x\right)-\eta \left(\hat{u}\right)\right]=0$$

Equation (16) indicates that the solution to the MLE can be found by mapping the data F(x) onto $F_{B}:=\left\{\eta \left(u\right):u\in R^{m}\right\}$ orthogonally to the tangent of $F_{A}:=\left\{\theta \left(u\right):u\in R^{m}\right\}$. As θ(u) and η(u) live in two different spaces $F_{A}$ and $F_{B}$, the inner product between dual spaces is defined as ${\langle \theta \left(u\right),\eta \left(u\right)\rangle }_{\Gamma }:=\theta {\left(u\right)}^{T}\cdot \Gamma \cdot \eta \left(u\right)$ with a metric Γ. For the flat manifold, the identity matrix serves as the metric Γ. By analogy with the MLE for the universal distribution given by (5), Hoeffding [14] presented another interpretation for the MLE of the curved exponential family. In the interpretation, $\eta (\hat{u})$ represents a point in $F_{B}$ which is located closest to the data point in the sense of the Kullback–Leibler divergence, i.e.,

(17) $$\hat{u}=\arg\min_{\eta \left(u\right)\in F_{B}}D\left[F\left(x\right)∥\eta_{u}\right]$$

where $D\left[F\left(x\right)∥\eta_{u}\right]$ denotes the Kullback–Leibler divergence from the multivariate joint distributions of F(x) to $\eta_{u}$.

Based on the above analysis, there are two important spaces related to a curved exponential family. One is called the natural parameter space, denoted by $\left\{\theta \right\}\subset A^{n}$, which denotes the enveloping space including all the distributions of exponential families, and the other is called the expectation parameter space, denoted by $\left\{\eta \right\}\subset B^{n}$, denoting the dual space of $A^{n}$. The two spaces are “dual” with each other and flat under the *e*-connection and *m*-connection, respectively. The curved exponential family (9) is regarded as submanifolds embedded in the two spaces, and the data can also be immersed in these spaces in the form of sufficient statistics F(x). Consequently, the estimators, such as the MLE given by (16), associated with the curved exponential families can be geometrically performed in the two spaces.

### 4.2. Geometric Demonstration of the Generalized Likelihood Ratio Test

As mentioned earlier, the GLRT is one of the most widely used approaches in composite hypothesis testing problems with unknown parameters in the PDFs. The data x have the PDF $p(x|u_{0};H_{0})$ under hypothesis $H_{0}$ and $p(x|u_{1};H_{1})$ under hypothesis $H_{1}$, where $u_{0}$ and $u_{1}$ are unknown parameters under each hypothesis. The GLRT enables a decision by means of replacement of the unknown parameters by their maximum likelihood estimates (MLEs) to implement a likelihood ratio test. The GLRT decides $H_{1}$ if

(18) $$L_{G}\left(x\right)=\frac{p(x|{\hat{u}}_{1};H_{1})}{p(x|{\hat{u}}_{0};H_{0})}>\gamma$$

where ${\hat{u}}_{i}$ is the MLE of $u_{i}$ (by maximizing $p(x|u_{i};H_{i})$).

From the perspective of information geometry, the probability distribution $p(x|u_{i};H_{i})$ is an element of the parameterized family of PDFs S={p(x|u),u∈Ω}, where $\Omega \subset R^{m}$ is the parameter set. For the curved exponential family S={p(x|u)}, it can be regarded as a submanifold embedding in the natural parameter space $\left\{\theta \right\}\subset A^{n}$, which includes all the distributions of exponential families. The curved exponential family *S* can be represented by a curve (or surface) {θ=θ(u)} embedded in the enveloping space $A^{n}$ by the nonlinear mapping u⟶θ(u). The expectation parameter space $\left\{\eta \right\}\subset B^{n}$ of *S* is a dual flat space to the natural parameter space {θ}, while the “realizations” of sufficient statistics F(x) of the distribution p(x|θ) can be immersed in this space. Consequently, the MLE is performed in the space $B^{n}$ by mapping the samples F(x) onto the submanifold specified by {η=η(u)} under the *m*-projection.

**Figure 2:**
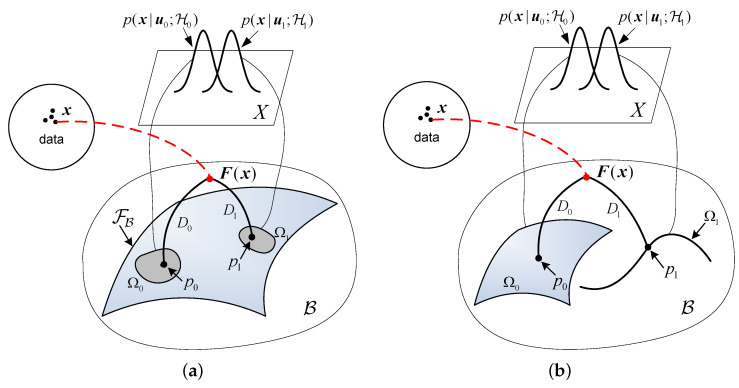
Geometry of the GLRT. (**a**) The case for the same unknown parameters under two hypotheses. (**b**) The case for different unknown parameters and different dimensionalities under two hypotheses.

As the parameters $u_{0}$ and $u_{1}$, as well as their dimensionalities, may or may not be the same under the null and alternative hypotheses, two pictures of the GLRT are presented for the two cases: one is with the same unknown parameters under each hypotheses and the other is with different parameters or different dimensionalities. The picture for the first case is illustrated in Figure 2a. In this case, distributions under two hypotheses share the same form and the same unknown parameter u. However, the parameter takes different value sets under different hypotheses. The family of S={p(x|u)} can be smoothly embedded as a surface $F_{B}$ specified by $\left\{\eta \left(u\right):u\in R^{m}\right\}$ in the space $B^{n}$. The hypotheses $p(x|u_{i};H_{i})$ with unknown $u_{i}$ define two “uncertainty volumes” $\Omega_{0}$ and $\Omega_{1}$ on the submanifold $F_{B}$. These volumes are the collections of probability distributions specified by the value sets of the unknown parameter $u_{i}$. The measurements x are immersed in $B^{n}$ in the form of sufficient statistics F(x). Consequently, the MLE can be found by “mapping” the samples F(x) onto the uncertainty volumes $\Omega_{0}$ and $\Omega_{1}$ on $F_{B}$. The points $p_{0}$ and $p_{1}$ in Figure 2 are the corresponding projections, i.e., the MLEs of the unknown parameter under two hypotheses. As indicated in (17), the MLEs can also be obtained by finding the points on $\Omega_{0}$ and $\Omega_{1}$ which are located closest to the data point in the sense of KLD, i.e.,

(19) $${\hat{u}}_{0}=\arg\underset{\eta \left(u\right)\in \Omega_{0}}{\inf}D\left[F\left(x\right)∥\eta_{u}\right],\;{\hat{u}}_{1}=\arg\underset{\eta \left(u\right)\in \Omega_{1}}{\inf}D\left[F\left(x\right)∥\eta_{u}\right]$$

and the corresponding minimum KLDs can be represented by

(20) $$D_{0}=D\left[F\left(x\right)∥p_{0}\right],\;D_{1}=D\left[F\left(x\right)∥p_{1}\right],$$

respectively.

It should be emphasized that the above “mapping” is a general concept. When the parameters to be estimated are not restricted by a given “value set”, the MLE is simply obtained by maximizing the likelihood and the projections will fall onto the submanifold $F_{B}$. However, if the parameters to be estimated are restricted in a given “value set”, the MLE should be operated by maximizing the likelihood with respect to the given parameter space. In the case where the projections fall outside the “uncertainty volumes”, the MLE solutions are given by those points which are closest to the data point described by (19).

Let $R(\eta_{0},\rho )$ be a *divergence sphere* centered at $\eta_{0}$ with radius ρ; that is, the submanifold of the enveloping space $B^{n}$ consisting of points η for which the KLD $D(\eta_{0}∥\eta )$ is equal to ρ. Denote this divergence sphere by

(21) $$R\left(\eta_{0},\rho \right)=\left\{\eta \in B^{n}\;|\;D\left(\eta_{0}∥\eta \right)=\rho \right\}$$

**Figure 3:**
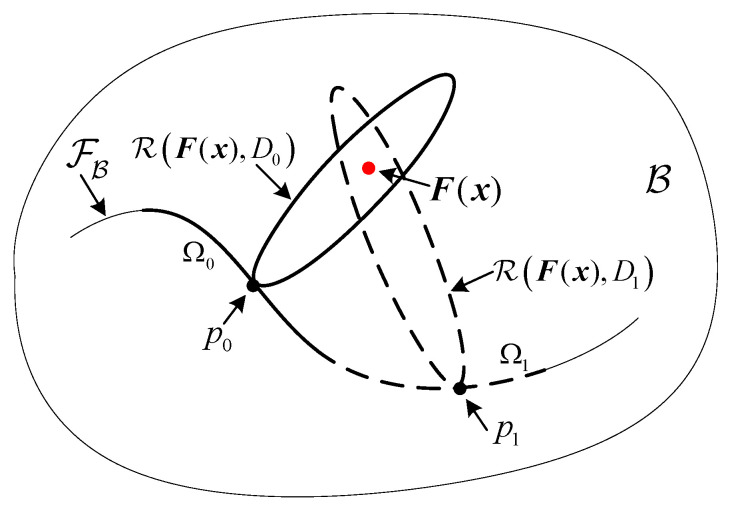
Illustration of the mapping via divergence spheres.

Then, the closest point in (19) may be more easily found via the divergence sphere with center F(x) and radius $D_{i}$ tangent to $\Omega_{i}$ at $p_{i}$, as illustrated in Figure 3. Consequently, according to (2), the GLRT can be geometrically performed by comparing the difference between the minimum KLDs $D_{0}$ and $D_{1}$ with a threshold $\gamma^{\prime }$, i.e.,

(22) $$D\left[F\left(x\right)∥p_{0}\right]-D\left[F\left(x\right)∥p_{1}\right]\underset{H_{0}}{\overset{H_{1}}{≷}}\gamma^{\prime }$$

In practice, the Neyman–Pearson criterion is commonly employed to determine the threshold $\gamma^{\prime }$ in (22) and the detector is of maximum probability of detection $P_{D}$ under a given probability of false alarm $P_{F}$. As a commonly used performance index, the missingprobability $P_{M}$ usually decays exponentially as the sample size increases. The rate of exponential decay can be represented by [22], as

(23) $$K≜\lim_{n\to \infty }-\frac{1}{N}\log P_{M}$$

Based on Stein’s lemma, for a constant false-alarm constraint, the best error exponent is related to the Kullback–Leibler divergence $D(p_{0}∥p_{1})$ from $p_{0}$ to $p_{1}$ [23], i.e.,

(24) $$K=D(p_{0}∥p_{1})$$

and

(25) $$P_{M}≐2^{-NK}$$

where ≐ denotes the first-order equivalence in the exponent. For example,

(26) $$a_{n}≐b_{n}\;\mathrm{means}\;\lim_{n\to \infty }\frac{1}{n}\log\frac{a_{n}}{b_{n}}=0.$$

In the above sense, the KLD from $p_{0}$ to $p_{1}$ is equivalent to the signal-to-noise ratio (SNR) of the underlying detection problem. Therefore, information geometry offers an insightful geometrical explanation for the detection performance of a Neyman–Pearson detector.

In the second case, the dimensionality of the unknown parameters $u_{0}$ and $u_{1}$ is different, while the dimensionality of the enveloping spaces is common for both hypotheses due to the same measurements x. However, the hypotheses may correspond to two separated submanifolds, $\Omega_{0}$ and $\Omega_{1}$, embedded in $B^{n}$ caused by the different dimensionality between the unknown parameters. As illustrated in Figure 2b, a surface and a curve are used to denote the submanifolds $\Omega_{0}$ and $\Omega_{1}$, corresponding to the two hypotheses, respectively. Similar to the first case, the GLRT with different unknown parameters may also be geometrically interpreted.

### 4.3. A Demonstration of One-Dimensional Curved Gaussian Distribution

Consider the following detection problem:

(27) $$\left\{\begin{array}{c} H_{0}:u<0\\ H_{1}:u>0 \end{array}\right.$$

The measurement originates from a curved Gaussian distribution

(28) $$x∼N(u,u^{2}a^{2})$$

where *a* is a positive constant and *u* is an unknown parameter.

The probability density function of the measurement is

(29) $$p\left(x|u\right)=\frac{1}{\sqrt{2\pi }a\left|u\right|}\exp\left[-\frac{{\left(x-u\right)}^{2}}{2a^{2}u^{2}}\right]$$

By reparameterization, the probability density function can be represented in the general form of a curved exponential family as

(30) $$\begin{array}{cc} p(x|u) & =\exp\left\{\frac{1}{a^{2}u}x-\frac{1}{2a^{2}u^{2}}x^{2}-\frac{1}{2a^{2}}-\ln\left(a\left|u\right|\right)-\frac{1}{2}\ln\left(2\pi \right)\right\}\\ \; & =\exp\left\{\ln a+\theta_{1}x+\theta_{2}x^{2}-\left[-\frac{\theta_{1}^{2}}{4\theta_{2}}-\frac{1}{2}\ln\left(-\theta_{2}\right)+\frac{1}{2}\ln\pi \right]\right\}\\ \; & =\exp\left\{C\left(x\right)+F_{1}\left(x\right)\theta_{1}+F_{2}\left(x\right)\theta_{2}-\varphi \left(\theta_{1},\;\theta_{2}\right)\right\} \end{array}$$

where C(x)=lna and the potential function φ is

(31) $$\varphi \left(\theta_{1},\;\theta_{2}\right)=-\frac{\theta_{1}^{2}}{4\theta_{2}}-\frac{1}{2}\ln\left(-\theta_{2}\right)+\frac{1}{2}\ln\pi$$

The above distributions with local parameter *u* correspond to a one-dimensional curved exponential family embedded in the natural parameter space A. The natural coordinates are

(32) $$\theta_{1}=\frac{1}{a^{2}u},\;\theta_{2}=-\frac{1}{2a^{2}u^{2}}$$

which defines a parabola (denoted by $F_{A}$)

(33) $$\theta_{2}=-\frac{a^{2}}{2}\theta_{1}^{2}$$

in A. The underlying distribution (28) can also be represented in the expectation parameter space B with expectation coordinates

(34) $$\eta_{1}=\frac{\partial \varphi }{\partial \theta_{1}}=u,\;\eta_{2}=\frac{\partial \varphi }{\partial \theta_{2}}=\left(a^{2}+1\right)u^{2}$$

which also defines a parabola (denoted by $F_{B}$)

(35) $$\eta_{2}=\left(a^{2}+1\right)\eta_{1}^{2}$$

in B.

The sufficient statistics F(x) obtained from samples x can be represented by

(36) $$F\left(x\right)={\left[x,x^{2}\right]}^{T}$$

**Figure 4:**
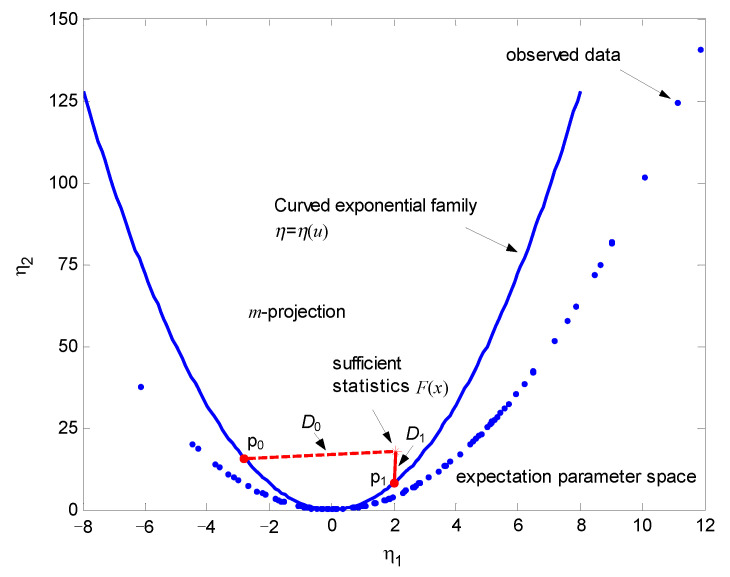
The geometric interpretation of the GLRT for one-dimensional curved Gaussian distribution.

Figure 4 shows the expectation parameter space and illustrates geometric interpretation of the underlying GLRT, where the blue parabola in the figure denotes embedding of the curved Gaussian distribution with parameter *u*. The submanifolds associated with two hypotheses can be geometrically represented by the blue parabolas (specified by $\eta_{1}<0$ and $\eta_{1}>0$, respectively). Without loss of generality, assume that a=1,u=2. The blue dots signify N=100 observations (measurements) in the expectation parameter space with the coordinates $\left(x,\;x^{2}\right)$. The statistical mean of the measurements are used to calculate the sufficient statistics F(x) which are denoted by a red asterisk. The MLEs of parameter *u* under two hypotheses are obtained by finding the points on the two submanifolds which are closest to the data point in the sense of KLD. According to (22), the GLRT can be geometrically performed by comparing the difference between the minimum KLDs $D_{0}$ and $D_{1}$ with a threshold $\gamma^{\prime }$.

### 4.4. Discussions

The geometric formulation of the GLRT presented above provides additional insights into the GLRT. To the best of our knowledge, there is no general analytical result associated with the performance of the GLRT in the literature [1]. The asymptotic analysis is only valid under the conditions that (1) the data sample size is large; and (2) the MLE asymptotically attains the Cramér-Rao lower bound (CRLB) of the underlying estimation problems.

It is known that the MLE with natural parameters is a sufficient statistic for an exponential family, and achieves the CRLB if a suitable measurement function is chosen for the estimation [8]. For the curved exponential families the MLE is not, in general, an efficient estimator, which means that the variance of MLE may not achieve CRLB with a finite number of samples. This indicates that when using a finite number of samples there will be a deterioration in performance for both MLE and GLRT when the underlying statistical model is a curved exponential family. There will be an inherent information loss (compared with the Fisher information) when implementingan estimation process if the statistical model is of nonlinearity. Roughly speaking, if the embedded submanifold $F_{B}$ in Figure 2a and $\Omega_{0}$, $\Omega_{1}$ in Figure 2b are curved, the MLEs will not achieve the CRLB due to the inherent information loss caused by the non-flatness of the statistical model. The information loss may be quantitatively calculated using the *e*-curvature of the statistical model [9].

Consequently, if the statistical model associated with a GLRT is not flat, i.e., the submanifolds shown in Figure 2 are curved, there will be a deterioration in performance for the GLRT using a finite number of samples. As sample size *N* increases, the sufficient statistics F(x) will be better matched to the statistical model and thus closer to the submanifolds (see Figure 2), and the divergence from data to the submanifold associated with the true hypothesis $H_{i}$ will be shorter. Asymptotically, as N→∞, the sufficient statistics will fall onto the submanifold associated with the true hypothesis $H_{i}$, so that the corresponding divergence $D_{i}$ reduces to zero. By then, the GLRT achieves a perfect performance.

## 5. Conclusions

In this paper, the generalized likelihood ratio test is addressed from a geometric viewpoint. Two pictures of the GLRT are presented in the philosophy of the information geometry theory. Both the detection and estimation associated with a GLRT are regarded as geometric operations on the manifolds of a parameterized family of probability distributions. As demonstrated in this work, the geometric interpretation of GLRT provides additional insights in the analysis of GLRT.

Potentially, more constructive analysis can be generalized based on the information geometry of GLRT. For example, the error exponent defined by (24) and (25) provides a useful performance index for the detection process associated with GLRT. When $p_{0}$ and $p_{1}$ in (24) are the estimates of an MLE (rather than the true values) of unknown parameters under each hypothesis, there may be a deterioration in performance in the estimation process. Determining how to incorporate such an “estimation loss” into the error exponent is an issue. Another open issue is the GLRT with PDFs of different forms for each hypothesis, which leads to a different distribution embedding associated with each hypothesis.

## Data Availability

Not applicable.
